# Unveiling underestimated species diversity within the Central American Coralsnake, a medically important complex of venomous taxa

**DOI:** 10.1038/s41598-023-37734-5

**Published:** 2023-07-19

**Authors:** Michael J. Jowers, Utpal Smart, Santiago Sánchez-Ramírez, John C. Murphy, Aarón Gómez, Renan J. Bosque, Goutam C. Sarker, Brice P. Noonan, J. Filipe Faria, D. James Harris, Nelson Jorge da Silva, Ana L. C. Prudente, John Weber, Philippe J. R. Kok, Gilson A. Rivas, Robert C. Jadin, Mahmood Sasa, Antonio Muñoz-Mérida, Gregorio Moreno-Rueda, Eric N. Smith

**Affiliations:** 1 CIBIO/InBIO (Centro de Investigação em Biodiversidade e Recursos Genéticos), Universidade do Porto, Campus Agrario De Vairão, 4485-661 Vairão, Portugal; 2 BIOPOLIS Program in Genomics, Biodiversity and Land Planning, CIBIO, Campus de Vairão, 4485-661 Vairão, Portugal; 3 Departamento de Zoología, Facultad de Ciencias, Universidad de Granada, 18071 Granada, Spain; 4 Department of Biology, Texas State University, 601 University Dr., San Marcos, TX 78666 USA; 5 Department of Ecology and Evolutionary Biology, University of Toronto, 25 Willcocks, Toronto, ON M5S 3B2 Canada; 6 Science and Education, Field Museum, 1400 S. Lake Shore Drive, Chicago, IL 60605 USA; 7 Facultad de Microbiología, Instituto Clodomiro Picado, Universidad de Costa Rica, San José, Costa Rica; 8 Department of Biological Sciences, Southwestern Oklahoma State University, Weatherford, OK 73096 USA; 9 Department of Biology and Amphibian and Reptile Diversity Research Center, University of Texas at Arlington, Arlington, TX 76019 USA; 10 Department of Biology, Cottey College, 1000 W. Austin Blvd, Nevada, MO 64772 USA; 11 Department of Biology, The University of Mississippi, Oxford, MS 38677 USA; 12 Departamento de Biologia, Faculdade de Ciências da Universidade do Porto, 4099-002 Porto, Portugal; 13 Pontifícia Universidade Católica de Goiás - Programa de Pós-Graduação em Ciências Ambientais e Saúde, Goiânia, Goiás 74605140 Brazil; 14 Laboratório de Herpetologia, Coordenação de Zoologia, Museu Paraense Emílio Goeldi (MPEG), Belém, Pará Brazil; 15 Programa de Pós-Graduação em Zoologia (UFPA/MPEG) and Biodiversidade e Evolução (MPEG), Belém, Pará Brazil; 16 Department of Geology, Grand Valley State University, Allendale, MI 49401 USA; 17 Department of Ecology and Vertebrate Zoology, Faculty of Biology and Environmental Protection, University of Łódź, 12/16 Banacha Str, 90-237 Lodz, Poland; 18 Department of Life Sciences, The Natural History Museum, London, SW7 5BD UK; 19 Museo de Biología, Facultad Experimental de Ciencias, Universidad del Zulia, Maracaibo, Venezuela; 20 Department of Biology and Museum of Natural History, University of Wisconsin Stevens Point, Stevens Point, WI 54481 USA; 21 Museo de Zoología, Centro de Investigación en Biodiversidad y Ecología Tropical, Universidad de Costa Rica, San José, Costa Rica

**Keywords:** Evolution, Evolutionary genetics, Phylogenetics, Speciation, Taxonomy

## Abstract

Coralsnakes of the genus *Micrurus* are a diverse group of venomous snakes ranging from the southern United States to southern South America. Much uncertainty remains over the genus diversity, and understanding *Micrurus* systematics is of medical importance. In particular, the widespread *Micrurus nigrocinctus* spans from Mexico throughout Central America and into Colombia, with a number of described subspecies. This study provides new insights into the phylogenetic relationships within *M. nigrocinctus* by examining sequence data from a broad sampling of specimens from Mexico, Guatemala, Honduras, Nicaragua, Costa Rica, and Panama*.* The recovered phylogenetic relationships suggest that *M. nigrocinctus* is a species complex originating in the Pliocene and composed of at least three distinct species-level lineages. In addition, recovery of highly divergent clades supports the elevation of some currently recognized subspecies to the full species rank while others may require synonymization.

## Introduction

The genus *Micrurus*, commonly known as coralsnakes, encompasses more than 85 species and is responsible for severe envenoming cases in the American continent^1–4^. Despite its medical importance, antivenom production is often deficient, due to the challenge of extracting enough venom and the difficulties of keeping coralsnakes in captivity^5,6^. Furthermore, the variable immunological cross-recognition variation between species poses an additional challenge for antivenom production by requiring antivenoms with an elevated degree of specificity^7–9^. Consequently, few laboratories manufacture *Micrurus* antivenoms, and several countries where the coralsnakes can be found lack the resources to mitigate the effects of envenomation^3^. Therefore, addressing the species diversity of the genus *Micrurus* remains of pivotal importance from both medical and conservation perspectives, as evidence suggests that the genetic diversity within the genus remains likely underestimated due to a lack of sampling throughout the species range^10,11^.

The lack of meristic traits in the genus *Micrurus* has resulted in many species being described primarily based on coloration^12,13^, resulting in the erroneous description of many regional color variants as species or subspecies within the genus^11,14^. Furthermore, their natural history, such as nocturnal and fossorial habits, has likely limited collection and resulted in insufficient comparative data. Therefore, it is not surprising that most, if not all, coralsnake groups need extensive taxonomic revisions (see^14^ for some taxonomic arrangements of Brazilian coralsnakes). Some studies have recovered well-established species complexes as paraphyletic^10,15,16^, and a recent study on the *M. diastema* species complex revealed a considerable level of mitochondrial introgression in Middle America^11^ and studies on Central, South and North American taxa showed high levels of intraspecific color polymorphism, traditionally resulting in an over-inflation of the number of recognized species^11,14,17,18^. The lack of systematic resolution of some groups of *Micrurus* has directly hindered the understanding of its evolutionary history, and only recently the first time-based phylogeographic study within the genus was published^10^. These studies highlight the difficulties of distinguishing and diagnosing coralsnake species and the state of *Micrurus* systematics.

The Central American coralsnake, *Micrurus nigrocinctus* (Girard, 1854)^19^, is the most studied coralsnake species probably due to its medical importance^1,20^ resulting from the highest number of envenomation cases of all New World elapids. This species has been recorded from Mexico’s Pacific versant in Chiapas, through Guatemala, Honduras, El Salvador, Nicaragua, Costa Rica, Panama, and into the Caribbean coast of Colombia. The *M. nigrocinctus* clade is monadal (i.e., with a body pattern of a single black body ring per red ring), with individuals often possessing a black snout, followed by a yellow or white parietal band and a black nuchal band; and red scales usually show some black pigment. The hemipenis is long, slender, and strongly bifurcated, with a hook-shaped apical spine on each lobe. Members of the clade tend to be medium-sized snakes with adults up to 750 mm. Males have supra-anal tubercles. Despite its medical importance and wide geographic distribution throughout Central America, *M. nigrocinctus* has a poorly resolved taxonomy.

Currently, there are six recognized subspecies of *M. nigrocinctus*, with various degrees of geographic distribution overlap: *M. n. babaspul* Roze 1967^21^, *M. n. coibensis* Schmidt 1936^22^, *M. n. divaricatus* (Hallowell 1854)^23^, *M. n. nigrocinctus* (Girard 1854)^19^, *M. n. zunilensis* Schmidt 1932^24^, and *M. n. ovandoensis* Schmidt & Smith 1943^25^. Recently, *Micrurus mosquitensis*, previously considered a subspecies of *M. nigrocinctus*, was given full species status^26^, but the relationship of this species to other members of the *M. nigrocinctus* group remains unresolved. Similarly, the phylogenetic relationship of *M. ruatanus*, a species from the island of Roatán that closely resembles *M. nigrocinctus* from mainland Honduras, remains unknown. Interestingly, the critically endangered *M. ruatanus*^27^ is the only *Micrurus* species found on an oceanic island, and can be considered an exception to a pattern of recent colonization since all other island coralsnakes are found on continental islands^10^. The island of Roatán is slightly peripheral to the continental shelf on the south flank of the Eocene-aged Cayman Trough oceanic spreading system^28^. Roatán is currently surrounded by water with depths of ≥ 2.5 km on its north flank and of ~ 550 m in the narrow passage to the west, linking it with the neighboring island of Utila and eventually the Honduran continental shelf (see geomapapp.org;^29^). It has been assumed that Pleistocene glacial sea-level drops would not have connected Roatán and the neighboring Bay Islands with the mainland^30,31^, suggesting that the ancestor of *M*. *ruatanus* colonized Roatán over water. Nonetheless, the subaerial terrains of Utila and Roatán are only 33 km apart today, and the bathymetry between Utila and the mainland is 25 m or less^29^. The unique distribution of *M. ruatanus* warrants investigating its phylogenetic relationships and species status since its taxonomy is also fraught with difficulties. Finally, due to its restricted distribution, *M. ruatanus* is considered critically endangered^27^, which increases the urgency to understand the evolutionary relationships in this group.

Given the aforementioned biomedical importance, the conservation status of the species and, the unresolved taxonomy of the group, we hereby aim to: (1) assess the diversity and phylogenetic relationships of *Micrurus nigrocinctus* throughout most of its distribution (southern Mexico, Honduras, Nicaragua, Costa Rica and Panama); (2) evaluate the phylogenetic position of *M. ruatanus* as its presence on an oceanic island remains enigmatic for New World coralsnakes; and (3) estimate the timing of evolutionary divergence among key lineages to correlate them to past geological events in the region to shed light on the evolutionary history of the group.

## Methods and materials

### Specimens and markers

In our analyses, we used 43 samples of the *M. nigrocinctus* complex, including *M. mosquitensis, M. latifasciatus nuchalis*, the continental subspecies of *M. nigrocinctus* (*M. n. divaricatus, M. n. nigrocinctus* and *M. n. zunilensis*), and two *M. ruatanus* (a skin shed from a live specimen kept in captivity (MJ1507) and another from a museum sample (H-6968). Tissue samples were obtained from many sources, including live zoological collections and museums (CH [Circulo Herpetológico de Panamá], LSUMZ [Lousiana State University Museum of Zoology], MVUP [Muséo de Vertebrados de la Universidad de Panamá], SMF [Senckenberg Museum], UCR [Universidad de Costa Rica], UF [University of Florida], UNAH [Universidad Autónoma de Honduras, USNM [United States National Museum], UTA [University of Texas at Arlington]), and/or directly in the field. When specimens were obtained in the field (see Supplementary Table S1) and/or when euthanasia was necessary, the protocol and procedures employed (specimen capture, handling, and preservation) were ethically reviewed and approved by The University of Texas at Arlington Institutional Animal Care and Use Committee (IACUC office) (Title of protocol: Phylogeny of New and Old World Venomous Snakes; Protocol number: A07.031 to ENS; or “Studies of Reptiles and Amphibians” A08.025). Euthanasia involved an intraperitoneal injection of Pentobarbital, 0.1 ml/kg, or 10% chloretone cardiac injection, depending on specific country substance regulations. Field samples were preserved by snap freezing, kept dry (sheds or road kills), or preserved in lysis buffer, 100% EtOH or RNAlater (Thermo Fisher Scientific). All methods were performed in accordance with the relevant guidelines and regulations. The reported study is in accordance with ARRIVE guidelines. In total, we include 34 newly sequenced specimens (Table S1). DNA extraction protocol follows^32^.

To infer the phylogenetic relationships and reconstruct the timing of evolutionary divergence, we sequenced the mitochondrial protein-coding genes NADH subunit 4 (ND4, 666 bp) and cytochrome *b* (Cytb, 711 bp). Templates were sequenced on both strands, and the complementary reads were used to resolve rare, ambiguous base calls in Sequencher v.4.9. Sequences were aligned in Seaview v.4.2.11^33^ under ClustalW2^34^ default settings. Nucleotide translation into proteins had no stop codons. Primers are listed in Supplementary Table S2. The GenBank sequences generated for this study are given in Supplementary Table S1.

### Phylogenetic analyses

We used PartitionFinder v.2^35^ to choose the optimal partitioning strategy under a greedy search scheme^36^. The most appropriate substitution models for the Bayesian Inference (BI) and RAxML^37^ analyses were determined by the Bayesian information criterion (BIC). Phylogenetic inference was based on the ND4 and Cytb gene fragments (complete alignment length; 1377 bp). The best partition scheme for the ND4 and Cytb alignment supported a codon partition for all analyses (BI, RAxML) (see supplementary Table S3). We only used previously published sequences with known vouchers, locality, and confirmed identification.

Bayesian inference trees were built in MrBayes v. 3.2^38^ under substitution models with codon-based partitions, default priors, and Markov chain settings, and random starting trees. Each run consisted of four chains of 20,000,000 generations, sampled every 20,000 generations, discarding 25% of the trees as burn-in. We used the software RAxML v7.0.4^37^ to perform a maximum likelihood (ML) inference of the evolutionary relationships between our samples with default settings and codon partition (Supplementary Table S3). Tree inferences were performed through the CIPRES platform^39^. In both analyses, we assigned *Micruroides euryxanthus* as the outgroup^40^. Genetic* p*-distances of combined Cytb and ND4 were calculated in MEGA X^41^ under a pairwise partial deletion option.

### Fossilized birth death divergence times estimation

We estimated divergence times using the Sampled Ancestors (SA v2.0) package^42^ in BEAST 2 v2.6.7^43^ hosted on the CIPRES Science Gateway^39^. ND4 and Cytb were concatenated into a single locus to reflect the linked state of mitochondrial genes. We used the General Time Reversible + Gamma Site Model (GTR + Γ) of evolution on the data to accommodate possible rate variation among lineages with an uncorrelated log–normal relaxed clock model.

We used the Fossilized Birth–Death (FBD) model^44^ for the tree prior, which provides an alternative to the incoherent approach of specifying calibration constraints and the associated probability distributions assigned to interior nodes. The FBD model directly incorporates all fossil data in a Bayesian framework^44^ and requires priors for only four parameters, namely: speciation rate (λ), extinction rate (μ), fossil recovery rate (ψ), and proportion of sampled extant species (ρ). Prior values for λ and μ were adopted from previously published speciation and extinction rates for *Micrurus*^45^.

Based on the above four parameters the diversification rate was assigned an exponential prior with a mean value of 0.14. In order to reduce the occurrence of extreme values (i.e., 0 or 1) for the sampling proportion rate, a beta prior distribution with shape parameters α = 2.0 and β = 2.0 was assigned. Given the inherent challenge of assigning a prior for the turnover (or relative extinction) rate, we kept the default uniform prior for this parameter. The parameter ρ was set to a value of 0.58 since we had 43 of the currently considered 85 extant New World coralsnake species represented in our phylogeny. Both the hyperparameters for the clock models were assigned an exponential prior distribution, with the mean (ucldMean) set to a value of 10.0 and the standard deviation (ucldStdev) set to 1.0.

We rooted the tree prior to an origin time of 23.0 Mya based on the age of the oldest known elapid fossils^46,47^ and assigned a lognormal prior distribution. Using the Mean in Real Space option the mean was specified to have a value of 8.2 and standard deviation of 1.0. The offset was set to 14.80 (i.e., the age of the oldest fossil used for calibration). Stem *Micrurus* were constrained with a date of 14.8 (16.0–13.6) Mya using *Micrurus* sp. indet, a specimen from the Barstovian of Nebraska^48,49^, which is considered the oldest New World elapid fossil. A second calibration point, a fossil dated to 830 K (1.8–0.3) Mya, was used to constrain the split between *M. fulvius* and *M. tener* as it is considered the MRCA between the two *Micrurus* species^50,51^. The latter age estimate has also been independently corroborated by a recent molecular study investigating the demographic history of the *M. fulvius* species complex^17^. Both fossil calibrations were included as blank sequences (represented by “?”) in the nexus alignment file and assigned uniform distribution priors (following^44^).

We ran two independent analyses for 1 × 10^8^ generations, sampling every 1,000 generations. We used the program Tracer v2.6.7^52^ to confirm the stationarity of the MCMC, with acceptable effective sample sizes of the posterior (ESS > 200) for each estimated prior. For each independent analysis, we also executed a run that samples only from priors while ignoring sequence data, which allowed us to compare marginal prior distributions on each parameter and assess the informativeness of the priors. After discarding 25% of samples as burn-in, the trees and parameter estimates from the independent runs were combined using LogCombiner v2.6.7 for a total of 10,000 final trees. Because the phylogenetic placement of the fossils was not meaningful, given that it was only informed by the prior (i.e., no associated sequence data), we removed them from the posterior trees using the FulltoExtantTreeConverter application in BEAST 2. Next, we used the program TreeAnnotator v2.6.7 to summarize parameter values of the samples from the posterior on a maximum clade credibility tree with mean node heights. The final tree was visualized and modified in FigTree v1.4.4^53^. The tree figures were generated in Inkscape Project (2020) (retrieved from https://inkscape.org).

## Results

### Phylogenetic relationships

**Figure 1:**
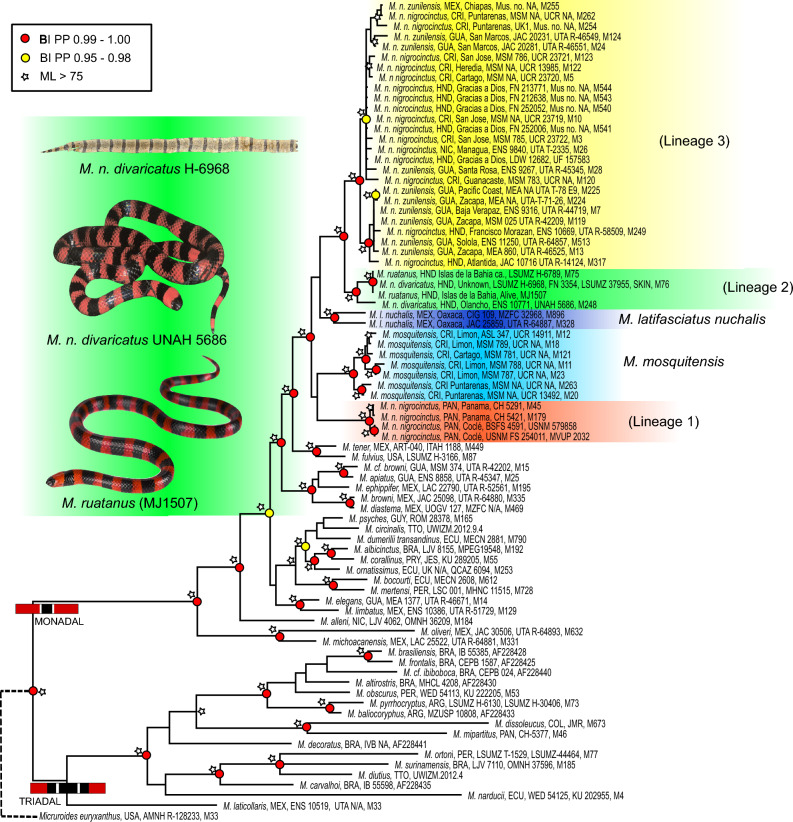
Maximum likelihood tree recovered from the RAxML analyses for the Cytb and ND4 gene fragments. Maximum likelihood bootstraps (> 75%) are indicated by nodes as white stars. Bayesian Inference node supports are shown with red and yellow circles. Photo credits: *M. ruatanus* (Jaime Culebras), *M. nigrocinctus divaricatus* skin (Jackson Roberts), *M. n. divaricatus* live specimen (Eric N. Smith).

This work is aligned with previous phylogenetic hypotheses of two primary sister clades of *Micrurus*: a triadal and a monadal clade^15,16,40,54^, with slight differences likely due to taxonomic coverage. Our phylogenetic tree recovers the triadal clade, constituted largely by South American species, and a monadal clade that includes species from South and Central America (Fig. 1).

Our results indicate that *Micrurus nigrocinctus* is polyphyletic due to the strongly supported inclusion of the clades constituted by *M. latifasciatus nuchalis* and *M. ruatanus* + *M. n. divaricatus*. All specimens of *M. n. nigrocinctus* from Panama (lineage 1) form a highly supported monophyletic group sister to *M. mosquitensis* from Costa Rica. This Isthmian Central American coralsnake group is sister to all other *M. nigrocinctus* ssp*.* represented in our work, including *M. l. nuchalis*. Within this group of mostly Nuclear Central American snakes, *M. l. nuchalis* is sister to the rest, including the well-supported clade formed by *M. ruatanus* + *M. n. divaricatus* (lineage 2) and its sister clade of all remaining *M. n. nigrocinctus* + *M. n. zunilensis* (lineage 3). The *M. n. nigrocinctus* + *M. n. zunilensis* clade is strongly supported by specimens from Southern Mexico to Costa Rica (Fig. 1).

### Genetic divergence

As could be expected for intraspecific genetic differentiation, the recovered genetic divergence within each of the *M. n. nigrocinctus* clades was low. Divergence within the larger clade of *M. n. zunilensis* + *M. n. nigrocintus* (lineage 3) was 1.1%, within Panama *M. n. nigrocintus* (lineage 1) 2.2%, within *M. mosquitensis* 1.3%, and within the clade containing *M. ruatanus* 1.1%. Both *M. ruatanus* samples from Islas de Bahia (Roatán, Honduras) and the *M. n. divaricatus* skin sample (unknown locality) recovered the same haplotype. In contrast the genetic differentiation of these and *M. n. divaricatus* from mainland Honduras (Olancho) was 1.8% (lineage 2) (Supplementary Table S4).

The highest genetic divergence between clades within the *M. nigrocinctus* complex was between the Panamanian *M. n. nigrocintus* and other clades, (to *M. mosquitensis* 5.5%, to *M. ruatanus* 5.5%, to *M. n. nigrocintus* + *M. n. zunilensis* [lineage 3] 5.5%). Divergence between *M. latifasciatus nuchalis* and other clades were also above 5% (to *M. ruatanus* 5%, to *M. mosquitensis* 5.6%, to lineage 3 it was 5.3%, to Panama *M. nigrocinctus* 5.7%). Divergence between *M. l. nuchalis* samples was 3.8%, higher than within any other lineage clade. Divergences between *M. ruatanus* and other clades were also moderately high (to *M. mosquitensis* 5.2%, to lineage 3, 3.2%) (Supplementary Table S4).

### Divergence time estimations

**Figure 2:**
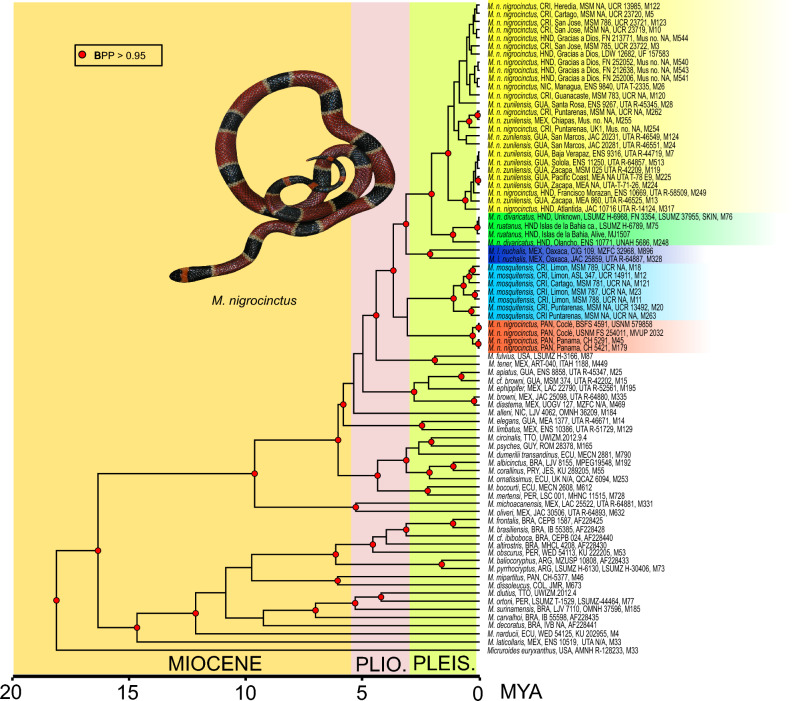
Bayesian time tree as inferred by BEAST 2.6.3 for the dataset of the Cytb and ND4 gene fragments for New World coralsnakes. Red nodes have Bayesian posterior probabilities > 0.95. Photo credits: *M. nigrocinctus* (Eric N. Smith).

Our analyses indicate that the divergence of *Micruroide*s and *Micrurus* occurred around 18 Mya (95% HPD, 14.1–26.1) and the split between the monadal and triadal lineages occurred around 16.5 Mya (95% HPD, 11.6–23.8). The earliest split in the triadal clade dates to 14.9 Mya (95% HPD, 9.9–21.1), and the monadal clade is with a more recent divergence around 9.8 Mya (95% HPD, 6.3–14.4) (Fig. 2).

Within the monadal clade, the first split within the *M. nigrocinctus* complex (including *M. latifasciatus nuchalis*) is estimated to date back to 3.7 Mya (95% HPD, 2.4–5.5). The Panamanian *M. n. nigrocinctus* and *M. mosquitensis* have their first splits at 0.3 Mya (95% HPD, 0.08–0.6) and 1.1 Mya (95% HPD, 0.5–1.9) respectively, with the divergence between their lineages dating to 3.1 Mya (95% HPD, 1.9–4.7). The *M. l. nuchalis* clade (2.1 Mya, 95% HPD, 1.0–3.4) is sister to the *M. ruatanus* + *M. n. divaricatus* clade (1.4 Mya, 95% HPD, 0.6–1.9) and to the *M. n. zunilensis* + *M. n. nigrocinctus* clade (1.3 Mya, 95% HPD, 0.8–2.1). The divergence time between the *M. ruatanus* clade and the most distant *M. m. divaricatus* (from mainland Honduras) is estimated at 1.4 Mya (95% HPD, 0.6–1.9) (Fig. 2).

## Discussion

### Lineage 1, *Micrurus nigrocinctus* from Panama

**Figure 3:**
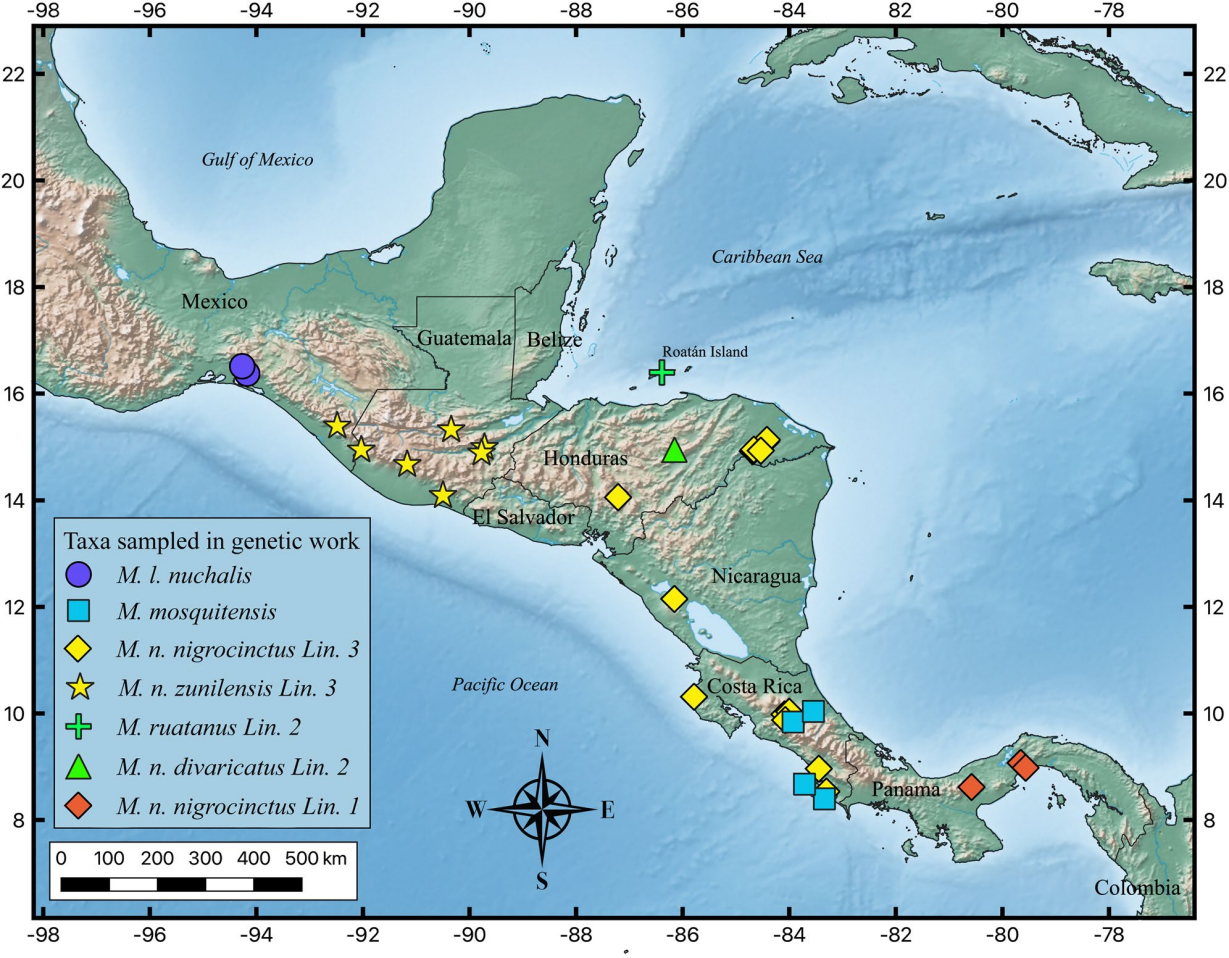
Map of Central America and all samples of *Micrurus* species with precise locality data and recovered within the *M. nigrocinctus* species complex. Map generated using QGIS version 3.24.2-Tisler (https://www.qgis.org/).

The well-supported Panamanian clade from our phylogenetic analysis strongly suggests that this group of snakes represents a separate evolutionary unit when compared to all other samples in the *M. nigrocinctus* species complex (Fig. 3). The oldest name available for the Panamanian group is *M. nigrocinctus* (Girard, 1854)^19^, a taxon with type locality in Taboga Island, a locality we have not sampled. Still, it is only 9.4 km from mainland Panama and on the Pacific discharge of the Panama Canal, in the Bay of Panama. Our molecular work and sampling efforts suggest that *M. nigrocinctus* is restricted to Panama, but we have a large geographical sampling gap between southwestern Costa Rica and Central Panama which might limit our conclusions. Including nuclear markers and comprehensive morphological analyses (beyond the current scope of this work) could improve our conclusion and corroborate the groups proposed by our analysis.

### Lineage 2, *Micrurus ruatanus* and *M. n. divaricatus*

Our phylogenetic analyses using mitochondrial data recover populations of *M. ruatanus* and *M. n. divaricatus* as indistinguishable members of the same lineage (lineage 2). *Micrurus n. divaricatus* as currently recognized, is restricted to a portion of mainland Honduras adjacent to Roatán island (Fig. 3). The taxonomic history of *Micrurus ruatanus* has been convoluted*,* initially described by Günther in 1895^55^ as *Elaps ruatanus*, it was later considered as *Elaps fulvius*^56^, a subspecies of *M. fulvius*^57^, a subspecies of *M. nigrocinctus*^58^, and again as a full species three decades after its original description^59^. Then, almost five decades later, the Roatán island population was again associated as a subspecies to mainland *M. nigrocinctus* by Roze^60^, as *M. n. ruatanus* (pp. 306, 332). Still within the same work it is given full species status (p. 311) and later (p. 333), it is questioned if it represents or not a distinct species. This recognition contradicts other works by the same author^21,61^ and other assessments of Honduran snakes (e.g.^62–64^) or coralsnakes in particular (e.g.,^12,13^).

*Micrurus ruatanus* is considered a distinctive species of coralsnake, possessing a bicolored head, body and tail of alternating black and red bands (somewhat orange when small), the nuchal band not reaching parietals, and 33–45 black rings on body^64^. *Micrurus ruatanus* is said to be different from bicolored and tricolored mainland Honduras *M. nigrocinctus* (including *M. n. divaricatus*) with the latter having fewer black body bands (30 or fewer, according to^64^) and females of the latter having fewer ventral scales (193–204 versus 206–224; males overlapping 183–194 versus 178–191 in *M. ruatanus*)^64^. Nonetheless, these differences seem minimal to separate them as different species, suggesting local differences likely conditioned through insular adaptation.

New World coralsnakes show considerable levels of introgression, as shown through ddRAD sequence data when compared to mtDNA phylogenies^11^, a limitation that could not be assessed in our mtDNA phylogeny. The taxonomy of these species raises interesting questions regarding the species´ morphology and venom evolution. The venom of *Micrurus ruatanus* is one of the most toxic among Central and South American snakes^65^ and has been compared to the Costa Rican *M. nigrocinctus* proteome^65^, but further work is needed to assess its systematics and taxonomy. The *M. ruatanus* sequenced for this study (specimen MJ1507) is the same individual for which the venom proteome was characterized^65^. Our molecular clock calibration suggests a possible evolutionary time of less than 1.4 Mya of venom evolution from mainland *M. n. divaricatus*. This time frame for venom evolution could be key to examining venom toxicity in the genus and deepening our temporal understanding of venom proteome change.

The island of Roatán has several endemic reptiles, some of which also occur on nearby islets. The endemics include *Anolis roatanensis*, *Ctenosaura oedirhina, Marisora roatanae, Norops roatanensis*, *Oxybelis wilsoni*, *Phyllodactylus palmeus, Sphaerodactylus leonardovaldesi*, *Sphaerodactylus rosaurae* and even a mammal (the Roatán Agouti, *Dasyprocta ruatanica*)^66^. Stepping-stone colonization likely emerged from the Honduran islands of Cayos Cochinos and/or the island of Utila, situated on the continental shelf surrounded by shallow waters of about 30–55 m. The island chain would have been connected to the mainland during the Pleistocene, between 13,000 and 18,000 years ago, at the end of the Wisconsin glacial period^31^, indicating recent isolation^67^. Therefore, *M. ruatanus* might have used the same colonization routes to reach Roatán in the Pliocene–Pleistocene, when the transmarine crossing would have been as little as 20 km. Nevertheless, Roatán Island was likely never connected to the mainland. The genetic divergence between the mainland and island form suggests genetic divergence dating to no earlier than the mid-Pleistocene.

### Lineage 3, *Micrurus n. c *and *M. n. zunilensis*

Within lineage 3 we recovered two-supported clades, each containing individuals belonging to *M. n. nigrocinctus* and *M. n. zunilensis.* One of these clades was constituted by several *M. n. zunilensis* from Guatemala and two individuals from Honduras belonging to *M. n. nigrocinctus*. The second clade encompassed specimens of *M. n. zunilensis* from Chiapas (Mexico) and Guatemala and specimens of *M. n. nigrocinctus* from Honduras, Nicaragua, and Puntarenas, Costa Rica. The two haplotype groups interdigitate populations along the Pacific of Guatemala and in central and northern Honduras. The resulting over-inflation of species can be avoided by validating the molecularly delimited species through independent lines of evidence (i.e., multiple data types). Therefore, statistical-morphological and genetic-multilocus studies must delimit this species geographically and morphologically.

### *Micrurus mosquitensis*

*Micrurus nigrocinctus mosquitensis* was described from Limón, Costa Rica, by Schmidt in 1933^59^ and treated as a subspecies for almost 70 years, until it was given full species status in 2004 by Solorzano^26^. Our phylogenetic analyses, including Pacific and Atlantic populations, suggest this taxon as distinct and the Pacific and Atlantic versant populations as belonging to the same taxon (Fig. 3). Using molecular data we confirm the arrangement^26^, contrary to McCranie^64^.

### Divergence estimates

The timings suggest that the New World coralsnakes emerged at the onset of the Langhian period. *Micrurus* dates to circa 16 Mya, when both monadal and triadal clades split. The triadal clade dates circa 15 Mya while the younger monadal clade likely dates to circa 10 Mya (Fig. 2). Overall, these fossil-based time estimates to infer timing for the whole genus remain congruent to other studies based on fossil estimates^16,45,68–70^ in comparison to when they are inferred with other dating methods^10,71^. Although our time estimates for the triadal clade are similar to those of Lee et al.^45^ and Zaher et al.^68,69^, they remain discordant to those of Hurtado Gomez et al.^70^. In addition, in their study^70^ they recover a more ancestral branching of the monadal rather than the triadal clade, in contradiction with other published works (e.g.,^69^). Their non-inclusion of the triadal snake *M. laticollaris* in their study likely influenced their tree topology. Similarly, the monadal clade dating remains older in our analyses than in previous studies, though similar to two previous works^10,45^.

The divergence between species within the *M. nigrocinctus* group falls within the Late Pliocene and Early Pleistocene, suggesting a time of climatic and environmental changes favorable to speciation events in the region. The low mtDNA genetic divergence between Roatán *M. ruatanus* and northern Honduras *M. n. divaricatus* suggests recent connections between localities, most likely through a recent Pleistocene dispersal event. Until this study, only *M. diutius* had been sequenced from an island (Trinidad) and compared to their counterpart in Guyana, recovering no genetic divergence for the ND4 locus^10^. This study further increases our understanding of colonization processes of New World coralsnakes on islands and in this instance, points to a recent connection due to a combination of low glacial sea levels during the Pleistocene and island hopping across deep (~ 500 m), but narrow (~ 20–40 km) ocean channels.

It must be noted that a certain degree of discrepancy is expected between results generated by different studies, given the differences in the sampling of genes, taxa, calibration types (fossil vs secondary), and notably the choice of priors for key parameters in BEAST. Additionally, node ages estimated under the FBD model are typically incongruent with those resulting from traditional node dating analyses as consistently shown by several recent studies^45,72,73^.

### Biomedical implications

Most of the published works on the composition and toxicity of the venom in *Micrurus nigrocinctus* come from specimens from the Central Plateau of Costa Rica (e.g.,^3,20,74,75^). In contrast, studies on the toxicity and composition of the venom of *M. nigrocinctus* in Panama are lacking. However, some preliminary chromatographic profiles suggest a high similarity with venoms of *M. n. nigrocinctus* from Costa Rica (data not shown) and some preliminary data from the research center and information on medicines and toxins (Centro de Investigación e Información en Medicamentos y Tóxicos, personal communication) of Panama confirm patient recovery from *M. nigrocintus* bites with Costa Rican antivenom. Fortunately, the most extensive geographic range within the *M. nigrocinctus* group is occupied by our lineage 3, which also occupies Central Costa Rica, suggesting that all of the antivenoms produced could have applicability for bites throughout Central America. *Micrurus* venoms can be classified as either PLA2-rich or 3FTx-rich^74,76^ and cross-species antivenom efficacy is highly dependent on the compositional predominance of either. *Micrurus nigrocinctus* from lineage 3, the most important coralsnakes from the medical point of view in Central America, has a venom rich in PLA2^77^ and the anticoral antivenom manufactured in Costa Rica uses this venom as an immunogen. This makes this antivenom mainly effective against snake venoms rich in PLA2, although it is less effective against those rich in 3FTx^1,78^. For example, both the venom of *M. mosquitensis*, a Costa Rican species sympatric with *M. n. nigrocinctus* lineage 3, predominantly rich in PLA2^8^, as well as that of *M. ruatanus*, predominantly rich in 3FTx, are neutralized by the antivenom produced in Costa Rica^65^.

### Current limitations and further taxonomical implications

The findings of this study are based on mitochondrial derived data and future research on these clades would greatly benefit from the addition of informative nuclear data. Unfortunately, nuclear markers for this study proved problematic to sequence and those that amplified were too conservative to resolve with confidence phylogenetic inferences. Therefore, the nuclear data were not included in the final analyses to avoid the improper statement that the phylogeny was derived from both genomes. Fortunately, a new ML tree RAD-seq data (Renan J. Bosque personal communication) derived from some of the exact same *Micrurus* individuals used for the mitochondrial DNA tree of this study is fully congruent with the recovered phylogenetic positions of lineages 1, 2 and 3, and therefore suggests that the mitochondrial tree is a species tree. Future phylogenetic congruence (may it be from morphological, -RAD-seq or nuclear sequence data) will suggest the need for taxonomical changes of these lineages.

### Conclusion

Our results strongly highlight divergent mtDNA clades within the *M. nigrocinctus* species complex. The evidence supported by morphological and/or nuclear gene-based evidence will likely elevate them to species level. *Micrurus nigrocinctus* from Panama is monophyletic and highly divergent from all other lineages. Using genetics we confirm the already elevated species *M. mosquitensis*^26^ in Costa Rica. *Micrurus ruatanus* seems restricted to Honduras and closely related to *M. n. divaricatus* from the mainland*,* with very low genetic differentiation. A group of currently recognized *M. n. nigrocinctus* and *M. n zulinensis* are recovered as monophyletic, with low genetic divergence despite their broad distribution from southern Mexico to Costa Rica, including Guatemala, Honduras, and Nicaragua. Furthermore, the phylogenetic placement of the two samples of *M. latifasciatus nuchalis* within the *M. nigrocinctus* species complex confirms the paraphyly of the group and therefore the need for a taxonomic rearrangement. Our findings have pivotal implications for future antivenom research, but we refrain from performing taxonomic changes until more data can be assessed for this complex of taxa.

## Supplementary Information

Supplementary Tables.

## Data Availability

The datasets generated and/or analyzed during the current study are available in the GenBank repository (https://www.ncbi.nlm.nih.gov/genbank/).
