# Supplementary material for: Unveiling underestimated species diversity within the Central American Coralsnake, a medically important complex of venomous taxa
Source: Sci Rep. 2023 Jul 19;13:11674. doi: 10.1038/s41598-023-37734-5 (PMC10356846; doi:10.1038/s41598-023-37734-5)
Supplement: Supplementary file 1 — Supplementary Tables. [file 41598_2023_37734_MOESM1_ESM.docx]

**SUPPLEMENTARY INFORMATION**

**Unveiling underestimated species diversity within the Central American Coralsnake, a medically important complex of venomous taxa**

Michael J. Jowers, Utpal Smart, Santiago Sánchez-Ramírez, John C. Murphy, Aaron Gómez, Renan J. Bosque, Goutam C. Sarker, Brice P. Noonan, J. Filipe Faria, D. James Harris, Nelson Jorge da Silva Junior, Ana L. C. Prudente, John Weber, Philippe J. R. Kok, Gilson A. Rivas, Robert C. Jadin, Mahmood Sasa, Antonio Muñoz-Mérida, Gregorio Moreno-Rueda, Eric N. Smith

**SI Table S1.** Specimens sequenced, taxonomy used in this paper, voucher information, and GenBank accession numbers.

| **Species Name** | **Museum No.** | **Field/Tissue No.** | **ENS Lab. No.** | **Country** | **Department/Province/State** | **Locality** | **Latitude** | **Longitude** | **Elevation (m)** | **Cytb** | **ND4** |
| --- | --- | --- | --- | --- | --- | --- | --- | --- | --- | --- | --- |
|  |  |  |  |  |  |  |  |  |  |  |  |
| *M. ruatanus* |  | MJ 1507 |  | Honduras | Islas de la Bahía | [ca. Isla de Roatán] | 16.395749 | -86.389428 | 0–50 | - | OP481977 |
| *M. ruatanus* | N/A | LSUMZ H-6789 | M75 | Honduras | Islas de la Bahía, ca. | [ca. Isla de Roatán] | 16.395749 | -86.389428 | 0–50 | OP514759 | OP481978 |
| *M. n. divaricatus* | UNAH 5686 | ENS 10771 | M248 | Honduras | Olancho | Municipio San Francisco de La Paz, Sierra de Agalta, El Aguacatal | 14.945000 | -86.153000 | 1176 | OP514761 | OP481980 |
| *M. n. divaricatus* | LSUMZ 37955 | LSUMZ H-6968 (FN 3354) | M76 | Honduras | Unknown |  |  |  |  | OP514760 | OP481979 |
| *M. latifasciatus nuchalis* | UTA R-64887 | JAC 25859 | M328 | Mexico | Oaxaca | Las Gradas^1^ | 16.511733 | -94.265631 | 217 | - | MG947691 |
| *M. latifasciatus nuchalis* | MZFC 32968 | CIG 109 | M896 | Mexico | Oaxaca | Tapanatepec^2^ | 16.372422 | -94.172000 | 76 | - | MG947634 |
| *M. n. nigrocinctus* | UCR 23720 | MSM N/A | M5 | Costa Rica | Cartago | La Union, Tres Ríos, San Ramón | 09.938880 | -84.006380 | 1365 | OP514779 | OP481995 |
| *M. n. nigrocinctus* | UCR | MSM 783 | M120 | Costa Rica | Guanacaste | Santa Rosa | 10.313370 | -85.786588 | 31 | MG947742 | MG947651 |
| *M. n. nigrocinctus* | UCR 13985 | MSM N/A | M122 | Costa Rica | Heredia | Santo Domingo [Santo Domingo Centro] | 09.983100 | -84.089400 | 1169 | OP514776 | OP481994 |
| *M. n. nigrocinctus* | SMF | UK 1 | M254 | Costa Rica | Puntarenas | Palmar Norte | 08.963675 | -83.445821 | 30 | OP514758 | - |
| *M. n. nigrocinctus* | UCR | MSM N/A | M262 | Costa Rica | Puntarenas | Peninsula de Osa, Corcovado [Puerto Jiménez] | 08.533868 | -83.303342 | 7 | OP514768 | - |
| *M. n. nigrocinctus* | UCR 23719 | MSM N/A | M10 | Costa Rica | San José | Desamparados (Desarmados in error), San Rafael Arriba | 09.876411 | -84.074825 | 1200 | - | OP481988 |
| *M. n. nigrocinctus* | UCR 23721 | MSM 786 | M123 | Costa Rica | San José | Moravia, San Jerónimo centro | 10.032084 | -84.000811 | 1436 | OP514771 | OP481992 |
| *M. n. nigrocinctus* | UCR 23722 | MSM 785 | M3 | Costa Rica | San José | Moravia Centro | 10.010133 | -84.011454 | 1405 | OP514769 | OP481990 |
| *M. n. zunilensis* | UTA R-44719 | ENS 9316 | M7 | Guatemala | Baja Verapaz | Carretera a Cobán, Km 137 | 15.319267 | -90.344533 | 1505 | OP514764 | OP481982 |
| *M. n. zunilensis* | UTA T-78-E9 | MEA N/A | M225 | Guatemala | Pacific Coast | Unknown | - | - | - | OP514762 | OP481981 |
| *M. n. zunilensis* | UTA R-46549 | JAC 20231 | M124 | Guatemala | San Marcos | Malacatán, Finca San Ignacio | 14.940800 | -92.031700 | 610–762 | OP514763 | MG947652 |
| *M. n. zunilensis* | UTA R-46551 | JAC 20281 | M24 | Guatemala | San Marcos | Malacatán, Finca San Ignacio | 14.940800 | -92.031700 | 610–762 | OP514765 | OP481983 |
| *M. n. zunilensis* | UTA R-45345 | ENS 9267 | M28 | Guatemala | Santa Rosa | Carretera Escuintla-Taxisco, km 102 | 14.089400 | -90.498600 | 254 | KU754317 | KU754457 |
| *M. n. zunilensis* | UTA R-64857 | ENS 11250 | M513 | Guatemala | Sololá | Cerro de Oro | 14.668000 | -91.170000 | 1620 | MG947784 | MG947713 |
| *M. n. zunilensis* | UTA R-42209 | MSM 025 | M119 | Guatemala | Zacapa | Teculután | 14.988375 | -89.719772 | 249 | OP514766 | MG947649 |
| *M. n. zunilensis* | UTA R-46525 | MEA 860 | M13 | Guatemala | Zacapa | El Arenal | 14.883833 | -89.775500 | 600 | KU754344 | KU754421 |
| *M. n. zunilensis* | UTA T-71-26 | MEA N/A | M224 | Guatemala | Zacapa | Unknown | - | - | - | OP514767 | OP481984 |
| *M. n. nigrocinctus* | UTA R-14124 | JAC 10716 | M317 | Honduras | Atlantida [?] | Mainland opposite of Hog Island [?] | - | - | - | OP514778 | - |
| *M. n. nigrocinctus* | UTA R-58509 | ENS 10669 | M249 | Honduras | Francisco Morazán | Tegucigalpa | 14.057300 | -87.211400 | 989 | MG947756 | MG947666 |
| *M. n. nigrocinctus* | N/A | FN 252052 | M540 | Honduras | Gracias a Dios | Rawa Kiamp | 15.100000 | -84.433333 | 84 | - | OP481987 |
| *M. n. nigrocinctus* | N/A | FN 252006 | M541 | Honduras | Gracias a Dios | Bachi Kiamp | 15.130000 | -84.420000 | 57 | - | OP481989 |
| *M. n. nigrocinctus* | SMF | FN 212638 | M543 | Honduras | Gracias a Dios | Warunta Tingni Kiamp | 14.916667 | -84.683333 | 150 | - | OP481986 |
| *M. n. nigrocinctus* | SMF | FN 213771 | M544 | Honduras | Gracias a Dios | Kipla Tingni Kiamp | 14.947800 | -84.665600 | 160 | - | OP481985 |
| *M. n. nigrocinctus* | UF 157583 | LDW 12682 |  | Honduras | Gracias a Dios | Río Tapalwás, Bodega | 14.927500 | -84.533889 | 134 | OP514772 | OP481974 |
| *M. n. zunilensis* | SMF | UK 2 | M255 | Mexico | Chiapas | Escuintla, Jamaica, Finca Zapote, ca. 15 km E Escuintla^1^ | 15.390466 | -92.481914 | 1000 | MG947759 | - |
| *M. n. nigrocinctus* | UTA T-2335 | ENS 9840 | M26 | Nicaragua | Managua | Instalaciones de MARENA (cerca de Aeropuerto Internacional, Ministerio de Ambiente y Recursos Naturales) | 12.150085 | -86.156407 | 52 | OP514770 | OP481991 |
| *M. n. mosquitensis* | UCR | MSM 781 | M121 | Costa Rica | Cartago | Cartago | 09.839047 | -83.927916 | 1346 | OP514783 | OP481997 |
| *M. n. mosquitensis* | UCR | MSM 788 | M11 | Costa Rica | Limón | Siquirres, Siquirres, Alto de Guayacán | 10.038900 | -83.547200 | 745 | OP514781 | - |
| *M. n. mosquitensis* | UCR | MSM 789 | M18 | Costa Rica | Limón | Siquirres, Siquirres, Alto de Guayacán | 10.038900 | -83.547200 | 745 | OP514785 | OP481998 |
| *M. n. mosquitensis* | UCR | MSM 787 | M23 | Costa Rica | Limón | Siquirres, Siquirres, Alto de Guayacán | 10.038900 | -83.547200 | 745 | OP514780 | OP481996 |
| *M. n. mosquitensis* | UCR 14911 | ASL 347 | M12 | Costa Rica | Limón | Siquirres, Siquirres, Alto de Guayacán | 10.038900 | -83.547200 | 745 | MG947741 | MG947650 |
| *M. n. mosquitensis* | UCR | MSM | M263 | Costa Rica | Puntarenas | Cantón de Osa, Bahia Drake, Punta San José, Marenco Lodge | 08.671780 | -83.714237 | 34 | OP514782 | - |
| *M. n. mosquitensis* | UCR 13492 | MSM | M20 | Costa Rica | Puntarenas | Golfito, Puerto Jiménez, Punta Piro | 08.396400 | -83.340800 | 10 | OP514784 | - |
| *M. n. nigrocinctus* | USNM 579858 | BSFS 4591 |  | Panama | Coclé | La Pintada, El Copé | 08.619400 | -80.579200 | 412 | OP514773 | OP481975 |
| *M. n. nigrocinctus* | CH 5291 |  | M45 | Panama | Panamá | Panama City, Albrook, Calle Boucet, casa 294 | 08.980556 | -79.564198 | 31 | OP514775 | OP481993 |
| *M. n. nigrocinctus* | CH 5421 |  | M179 | Panama | Panamá | Unknown [ca. P. N. Soberanía] | 09.072529 | -79.657907 | 80 | OP514777 | - |
| *M. n. nigrocinctus* | MVUP 2032 | USNM-FS 254011 |  | Panama | Coclé |  |  |  |  | OP514774 | OP481976 |
|  |  |  |  |  |  |  |  |  |  |  |  |
| **Outgroup** |  |  |  |  |  |  |  |  |  |  |  |
| *M. albicinctus* | MPEG 19548 | LJV 8155 | M192 | Brazil | Amazonas | 3 30.9' S, 59 54.2' W | -03.515000 | -59.903333 | 23 | - | JF308714 |
| *M. alleni* | OMNH 36209 | LJV 4062 | M184 | Nicaragua | Rio San Juan | ca 15 km S El Castillo on N bank Rio San Juan at Isla El Diamante | 10.933333 | -84.300000 | 107 | - | KX090913 |
| *M. altirostris* | MHCL 4208 |  |  | Brazil | Parana | Irati |  |  |  | - | AF228430 |
| *M. apiatus* | UTA R-45347 | ENS 8858 | M25 | Guatemala | Huehuetenango | Barillas Finca Chiblac | 15.885120 | -91.240670 | 930 | MG947757 | MG947667 |
| *M. baliocoryphus* | MZUSP 10808 |  |  | Argentina | Corrientes | Santa Rosa/Paso de la Patria |  |  |  | - | AF228433 |
| *M. bocourti* | MECN 2608 |  | M612 | Ecuador | Loja | Macará, Macará, Macara Reserva Jatumpamba | -04.656517 | -79.504555 | 2452 | - | KP998031 |
| *M. brasiliensis* | IB 55385 |  |  | Brazil | Bahia | Barreiras |  |  |  | - | AF228428 |
| *M. browni* | UTA R-64880 | JAC 25098 | M335 | Mexico | Guerrero | Sierra Madre del Sur, San Vicente de Benitez | 17.290610 | -100.279550 | 951 | MG947776 | MG947696 |
| *M. carvalhoi* | IB 55598 |  |  | Brazil | Mato Grosso | Rio Prieto |  |  |  | - | AF228435 |
| *M. cf. browni* | UTA R-42202 | MSM 374 | M15 | Guatemala | Huehuetenango | Nenton, Hacienda Miramar, Finca Los Cimientos (Grijalva Valley) | 15.834670 | -91.816330 | 694 | KU754313 | KU754414 |
| *M. cf. ibiboboca* | CEPB 024 |  |  | Brazil | Alagoas | Xingó |  |  |  | - | AF228440 |
| *M. circinalis* | UWIZM.2012.9.4 |  |  | Trinidad and Tobago | Trinidad | Driveway, Spinghill Estate 10° 41' 29.73" N, 61° 17' 15.99" W | 10.6915920 | -61.287775 | 221 | MK534162  (short sequence) | MK534172 |
| *M. corallinus* | KU 289205 | JES | M55 | Paraguay | Itapua | Parque Nacional San Rafael, 34 km NW Maria Auxiladora (26 21'S; 55 31'W) | -26.350000 | -55.516660 | 408 | - | JF308715 |
| *M. decoratus* | IVB NA |  |  | Brazil | Rio de Janeiro | Teresópolis |  |  |  | - | AF228441 |
| *M. diastema* | MZFC | UOGV 127 | M469 | Mexico | Veracruz | Las Choapas | 17.919574 | -94.104046 | 25 | - | MG947707 |
| *M. dissoleucus* |  | JMR | M673 | Colombia | Magdalena | Santa Marta, El Rodadero | 11.209649 | -74.221760 | 13 | - | JF308717 |
| *M. diutius* | UWIZM.2012.4. |  |  | Trinidad and Tobago | Trinidad | Gran Sual Trace, Mamal Main Road | 10.450306 | -61.279497 | 75 | MK534166 | MK534176 |
| *M. dumerilii transandinus* | MECN 2881 |  | M790 | Ecuador | Esmeraldas | Muisne, San Francisco, Río San Francisco | 00.699444 | -80.018057 | 80 | - | KP998035 |
| *M. elegans* | UTA R-46671 | MEA 1377 | M14 | Guatemala | Huehuetenango | Nentón, Aldea Yalambojogh, Lagunal Yolnabaj | 16.040223 | -91.578402 | 1130 | MG947745 | MG947655 |
| *M. ephippifer* | UTA R-52561 | JAC 22790 | M195 | Mexico | Oaxaca | Mpio. Teotitlan, Sierra Mazateca, San Bernardino | 18.138283 | -97.017650 | 2030 | - | MG947658 |
| *M. frontalis* | CEPB 1587 |  |  | Brazil | Goias | Guapo/Hidrolândia |  |  |  | - | AF228425 |
| *M. fulvius* | LSUMZ H-3166 |  | M87 | USA | Florida | Panhandle | 30.687148 | -86.110727 |  | KU754345 | KU754420 |
| *M. laticollaris* | MZFC/UTA | ENS 10519 | M33 | Mexico | Morelos | Cuernavaca, Zona Industrial CIVAC, Parque El Texcal | 18.899019 | -99.149367 | 1410 | - | KU754446 |
| *M. limbatus* | UTA R-51729 | ENS 10386 | M129 | Mexico | Veracruz | Sierra de los Tuxtlas: San Pedro Soteapan (Municipio) | 18.203052 | -94.867313 | 285 | MG947743 | MG947653 |
| *M. mertensi* | MHNC 11515 | LSC 001 | M728 | Peru | Cajamarca | Santa Cruz: Pulán: El Lanche: | -06.696517 | -78.959389 | 2000 | - | KP998032 |
| *M. michoacanensis* | UTA R-64881 | JAC 25522 | M331 | Mexico | Michoacan | Carretera Playa Azul-Manzanillo | 18.416010 | -103.53206 | 17 | MG947774 | MG947693 |
| *M. mipartitus* | CH-5377 |  | M46 | Panama | Cocle | La Mina, Distrito de Penonomé | 08.943820 | -80.274420 |  | EF137414 | EF137406 |
| *M. narducii* | KU 202955 | WED 54125 | M4 | Ecuador | Napo | 0.7 km S Arosemena Tola | -01.172669 | -77.856924 | 508 | - | EF137404 |
| *M. obscurus* | KU 222205 | WED 54113 | M53 | Peru | Loreto | 1.5 km N Teniente Lopez | -02.584123 | -76.115921 | 227 | - | JF308708 |
| *M. oliveri* | UTA R-64893 | JAC 30506 | M632 | Mexico | Colima | Road from Hway. 54 [110] to Ixtlahuacan | 19.029380 | -103.78786 | 255 | - | MG947722 |
| *M. ornatissimus* | QCAZ 6094 | UK | M253 | Ecuador | Zamora-Chinchipe | Zumba (Escuela Manuela Cañizares) | -04.865228 | -79.134590 | 1220 | - | KP998028 |
| *M. ortoni* | LSUMZ H-44464 | LSUMZ T-1529/ FN 294 | M77 | Peru | Loreto | Quebrada Orán, ca. 5 km N Río Amanzonas, 85 km N Iquitos | -03.457249 | -72.514121 | 100 | - | JF308704 |
| *M. psyches* | ROM 28378 |  | M165 | Guyana | Rupununi | Paramakatoi | 04.697220 | -59.786660 | 696 | - | JF308713 |
| *M. pyrrhocryptus* | LSUMZ H-30406 | LSUMZ T-6130/HCD 2510 | M73 | Argentina | Buenos Aires | Vicinity of Buenos Aires |  |  |  | - | JF308705 |
| *M. surinamensis* | OMNH 37596 | LJV 7110 | M185 | Brazil | Rondonia | Rio Formoso: Parque Estadual Guajara-Mirim, approx. 90 km N Nova Mamore; S 10 19', W 64 33' | -10.316667 | -64.550000 | 148 | EF137415 | EF137407 |
| *M. tener* | ITAH 1188 | ART- 040 | M449 | Mexico | Hidalgo | Municipio de Tepehuacan de Guerrero, camino a Teyahuala | 21.043974 | -98.856625 | 1342 | KU754294 | KU754431 |
| *Micruroides euryxanthus* | AMNH R-128233 |  | M41 | United States | Arizona | Cochise County |  |  |  | EF137416 | EF137408 |

Notes

^1^Wrong latitude and longitude in Reyes-Velasco et al. (2020)

^2^Wrong locality in Reyes-Velasco et al. (2020) GenBank submission

**Table S2.** Primers used in gene fragments amplification. Gene fragments amplified, primer and reference.

| **Gene** | **Primer name and sequence** | **Reference** |
| --- | --- | --- |
| cytb | 14910 5’- GACCTGTGATMTGAAAAACCAYCG -3' | Burbrink et al., (2002) |
| cytb | H16064 5’- CTTTGGTTTACAAGAACAATGCTT -3' | Burbrink et al., (2002) |
| nd4 | ND4 5’- CACCTATGACTACCAAAAGCTCATGTAGAAGC - 3’ | Arevalo et al., (1994) |
| nd4 | LEU 5’- CATTACTTTTACTTGGATTTGCACCA - 3’ | Arevalo et al., (1994) |

**References**

Arèvalo, E., Davis, S. K., & Sites, J. W. Jr. (1994). Mitochondrial DNA Sequence Divergence and Phylogenetic Relationships among Eight Chromosome Races of the *Sceloporus Grammicus* Complex (Phrynosomatidae) in Central Mexico, *Systematic Biology*, 43, 387–418

Burbrink, F. T., Lawson, R., & Slowinski, B. B. (2000). Mitochondrial DNA phylogeography of the polytypic North American rat snake (*Elaphe obsoleta*): a critique of the subspecies concept*. Evolution,* 54, 2107**–**2118.

**Table S3** Partition scheme results inferred from PartitionFinder2.

| **Partition** | **Model** | **Locus** |
| --- | --- | --- |
| 1 | GTR+I+G | Cytb codon 1, Nd4 codon 1 |
| 2 | GTR+I+G | Cytb codon 2, Nd4 codon 2 |
| 3 | GTR+G | Cytb codon 3, Nd4 codon 3 |

Table S4. *P*-distance matrix of combined Cytb and ND4 computed in MEGA X under a pairwise partial deletion option.

|  |  | 1 | 2 | 3 | 4 | 5 | 6 | 7 | 8 | 9 | 10 | 11 | 12 | 13 | 14 | 15 | 16 | 17 | 18 | 19 | 20 | 21 | 22 | 23 | 24 | 25 | 26 | 27 | 28 | 29 | 30 | 31 | 32 | 33 | 34 | 35 | 36 | 37 | 38 | 39 | 40 | 41 | 42 | 43 |
| --- | --- | --- | --- | --- | --- | --- | --- | --- | --- | --- | --- | --- | --- | --- | --- | --- | --- | --- | --- | --- | --- | --- | --- | --- | --- | --- | --- | --- | --- | --- | --- | --- | --- | --- | --- | --- | --- | --- | --- | --- | --- | --- | --- | --- |
| 1 | *M. n. divaricatus,* HND, Unknown, LSUMZ H-6968, FN 3354, LSUMZ 37955, SKIN, M76 | - |  |  |  |  |  |  |  |  |  |  |  |  |  |  |  |  |  |  |  |  |  |  |  |  |  |  |  |  |  |  |  |  |  |  |  |  |  |  |  |  |  |  |
| 2 | *M. n. divaricatus,* HND, Olancho, ENS 10771, UNAH 5686, M248 | 0,02 | - |  |  |  |  |  |  |  |  |  |  |  |  |  |  |  |  |  |  |  |  |  |  |  |  |  |  |  |  |  |  |  |  |  |  |  |  |  |  |  |  |  |
| 3 | *M. ruatanus,* HND, Islas de la Bahia, Alive, MJ1507 | 0,00 | 0,02 | - |  |  |  |  |  |  |  |  |  |  |  |  |  |  |  |  |  |  |  |  |  |  |  |  |  |  |  |  |  |  |  |  |  |  |  |  |  |  |  |  |
| 4 | *M. ruatanus,* HND, Islas de la Bahia ca., LSUMZ H-6789, M75 | 0,00 | 0,02 | 0,00 | - |  |  |  |  |  |  |  |  |  |  |  |  |  |  |  |  |  |  |  |  |  |  |  |  |  |  |  |  |  |  |  |  |  |  |  |  |  |  |  |
| 5 | *M. n. zunilensis*, MEX, Chiapas, Mus. No. NA, M255 | 0,02 | 0,02 | ? | 0,02 | - |  |  |  |  |  |  |  |  |  |  |  |  |  |  |  |  |  |  |  |  |  |  |  |  |  |  |  |  |  |  |  |  |  |  |  |  |  |  |
| 6 | *M. n. zunilensi*s, GUA, Pacific Coast, MEA NA UTA T-78 E9, M225 | 0,03 | 0,03 | 0,04 | 0,03 | 0,02 | - |  |  |  |  |  |  |  |  |  |  |  |  |  |  |  |  |  |  |  |  |  |  |  |  |  |  |  |  |  |  |  |  |  |  |  |  |  |
| 7 | *M. n. nigrocinctus*, HND, Gracias a Dios, FN 213771, Mus no. NA, M544 | 0,03 | 0,03 | 0,03 | 0,03 | ? | 0,01 | - |  |  |  |  |  |  |  |  |  |  |  |  |  |  |  |  |  |  |  |  |  |  |  |  |  |  |  |  |  |  |  |  |  |  |  |  |
| 8 | *M. n. nigrocinctus*, HND, Gracias a Dios, FN 212638, Mus no. NA, M543 | 0,03 | 0,03 | 0,03 | 0,03 | ? | 0,01 | 0,00 | - |  |  |  |  |  |  |  |  |  |  |  |  |  |  |  |  |  |  |  |  |  |  |  |  |  |  |  |  |  |  |  |  |  |  |  |
| 9 | *M. n. nicrocinctus*, HND, Gracias a Dios, FN 252052, Mus no. NA, M540 | 0,03 | 0,03 | 0,03 | 0,03 | ? | 0,01 | 0,00 | 0,00 | - |  |  |  |  |  |  |  |  |  |  |  |  |  |  |  |  |  |  |  |  |  |  |  |  |  |  |  |  |  |  |  |  |  |  |
| 10 | *M. n. nigrocinctus*, CRI, San Jose, MSM NA, UCR 2370, M10 | 0,04 | 0,03 | 0,04 | 0,04 | ? | 0,02 | 0,00 | 0,00 | 0,00 | - |  |  |  |  |  |  |  |  |  |  |  |  |  |  |  |  |  |  |  |  |  |  |  |  |  |  |  |  |  |  |  |  |  |
| 11 | *M. n. nigrocinctus*, HND, Gracias a Dios, FN 252006, Mus no. NA, M541 | 0,03 | 0,03 | 0,03 | 0,03 | ? | 0,01 | 0,00 | 0,00 | 0,00 | 0,00 | - |  |  |  |  |  |  |  |  |  |  |  |  |  |  |  |  |  |  |  |  |  |  |  |  |  |  |  |  |  |  |  |  |
| 12 | *M. n. nigrocinctus*, CRI, Puntarenas, UK1, Mus. No. NA, M254 | 0,03 | 0,02 | ? | 0,03 | 0,01 | 0,02 | ? | ? | ? | ? | ? | - |  |  |  |  |  |  |  |  |  |  |  |  |  |  |  |  |  |  |  |  |  |  |  |  |  |  |  |  |  |  |  |
| 13 | *M. n. nigrocinctus*, CRI, Puntarenas, MSM NA, UCR NA, M262 | 0,02 | 0,02 | ? | 0,02 | 0,00 | 0,01 | ? | ? | ? | ? | ? | 0,00 | - |  |  |  |  |  |  |  |  |  |  |  |  |  |  |  |  |  |  |  |  |  |  |  |  |  |  |  |  |  |  |
| 14 | *M. n. zunilensis*, GUA, San Marcos, JAC 20231, UTA R-46549, M124 | 0,03 | 0,03 | 0,04 | 0,03 | 0,01 | 0,02 | 0,02 | 0,02 | 0,02 | 0,02 | 0,02 | 0,01 | 0,01 | - |  |  |  |  |  |  |  |  |  |  |  |  |  |  |  |  |  |  |  |  |  |  |  |  |  |  |  |  |  |
| 15 | *M. n. zunilensis*, GUA, Baja Verapaz, ENS 9316, UTA R-44719, M7 | 0,03 | 0,03 | 0,04 | 0,03 | 0,01 | 0,00 | 0,01 | 0,01 | 0,01 | 0,02 | 0,01 | 0,01 | 0,01 | 0,02 | - |  |  |  |  |  |  |  |  |  |  |  |  |  |  |  |  |  |  |  |  |  |  |  |  |  |  |  |  |
| 16 | *M. n. nigrocinctus*, CRI, San Jose, MSM 785, UCR 23722, M3 | 0,03 | 0,03 | 0,04 | 0,03 | 0,01 | 0,02 | 0,00 | 0,00 | 0,00 | 0,00 | 0,00 | 0,02 | 0,01 | 0,02 | 0,02 | - |  |  |  |  |  |  |  |  |  |  |  |  |  |  |  |  |  |  |  |  |  |  |  |  |  |  |  |
| 17 | *M. n. zunilensis*, GUA, San Marcos, JAC 20281, UTA R-46551, M24 | 0,03 | 0,03 | 0,03 | 0,03 | 0,01 | 0,02 | 0,00 | 0,00 | 0,00 | 0,00 | 0,00 | 0,01 | 0,01 | 0,01 | 0,02 | 0,01 | - |  |  |  |  |  |  |  |  |  |  |  |  |  |  |  |  |  |  |  |  |  |  |  |  |  |  |
| 18 | *M. n. nigrocinctus*, NIC, Managua, ENS 9840, UTA T-2335, M26 | 0,03 | 0,03 | 0,03 | 0,03 | 0,02 | 0,02 | 0,00 | 0,00 | 0,00 | 0,00 | 0,00 | 0,02 | 0,02 | 0,02 | 0,02 | 0,01 | 0,01 | - |  |  |  |  |  |  |  |  |  |  |  |  |  |  |  |  |  |  |  |  |  |  |  |  |  |
| 19 | *M. n. nigrocinctus*, CRI, San Jose, MSM 786, UCR 23721, M123 | 0,04 | 0,04 | 0,04 | 0,04 | 0,02 | 0,02 | 0,01 | 0,01 | 0,01 | 0,01 | 0,01 | 0,02 | 0,02 | 0,02 | 0,02 | 0,01 | 0,01 | 0,01 | - |  |  |  |  |  |  |  |  |  |  |  |  |  |  |  |  |  |  |  |  |  |  |  |  |
| 20 | *M. n. nigrocinctus*, CRI, Guanacaste, MSM 783, UCR NA, M120 | 0,04 | 0,04 | 0,04 | 0,04 | 0,02 | 0,02 | 0,01 | 0,01 | 0,01 | 0,01 | 0,01 | 0,03 | 0,02 | 0,02 | 0,02 | 0,01 | 0,01 | 0,01 | 0,01 | - |  |  |  |  |  |  |  |  |  |  |  |  |  |  |  |  |  |  |  |  |  |  |  |
| 21 | *M. n. zunilensis*, GUA, Zacapa, MSM 025 UTA R 42209, M119 | 0,03 | 0,03 | 0,04 | 0,03 | 0,01 | 0,00 | 0,02 | 0,02 | 0,02 | 0,02 | 0,02 | 0,01 | 0,01 | 0,02 | 0,00 | 0,02 | 0,02 | 0,02 | 0,02 | 0,02 | - |  |  |  |  |  |  |  |  |  |  |  |  |  |  |  |  |  |  |  |  |  |  |
| 22 | *M. n. nigrocinctus*, CRI, Heredia, MSM NA, UCR 13985, M122 | 0,03 | 0,03 | 0,03 | 0,03 | 0,02 | 0,02 | 0,00 | 0,00 | 0,00 | 0,00 | 0,00 | 0,02 | 0,02 | 0,02 | 0,02 | 0,01 | 0,01 | 0,01 | 0,00 | 0,01 | 0,02 | - |  |  |  |  |  |  |  |  |  |  |  |  |  |  |  |  |  |  |  |  |  |
| 23 | *M. n. nigrocinctus*, HND, Atlantida, JAC 10716 UTA R-14124, M317 | 0,02 | 0,03 | ? | 0,02 | 0,02 | 0,01 | ? | ? | ? | ? | ? | 0,02 | 0,02 | 0,02 | 0,01 | 0,02 | 0,02 | 0,03 | 0,02 | 0,03 | 0,01 | 0,02 | - |  |  |  |  |  |  |  |  |  |  |  |  |  |  |  |  |  |  |  |  |
| 24 | *M. n. zunilensis*, GUA, Zacapa, MEA NA, UTA-T-71-26, M224 | 0,03 | 0,03 | 0,04 | 0,03 | 0,01 | 0,00 | 0,02 | 0,02 | 0,02 | 0,02 | 0,02 | 0,01 | 0,01 | 0,02 | 0,00 | 0,02 | 0,01 | 0,02 | 0,02 | 0,02 | 0,00 | 0,02 | 0,01 | - |  |  |  |  |  |  |  |  |  |  |  |  |  |  |  |  |  |  |  |
| 25 | *M. n. nigrocinctus*, CRI, Cartago, MSM NA, UCR 23720, M5 | 0,03 | 0,03 | 0,04 | 0,03 | 0,02 | 0,02 | 0,00 | 0,00 | 0,00 | 0,00 | 0,00 | 0,02 | 0,02 | 0,02 | 0,02 | 0,01 | 0,01 | 0,01 | 0,00 | 0,01 | 0,02 | 0,00 | 0,02 | 0,02 | - |  |  |  |  |  |  |  |  |  |  |  |  |  |  |  |  |  |  |
| 26 | *M. n. nigrocinctus*, HND, Francisco Morazan, ENS 10669, UTA R-58509, M249 | 0,03 | 0,03 | 0,04 | 0,03 | 0,02 | 0,00 | 0,02 | 0,02 | 0,02 | 0,02 | 0,02 | 0,02 | 0,02 | 0,02 | 0,00 | 0,02 | 0,02 | 0,02 | 0,02 | 0,02 | 0,00 | 0,02 | 0,01 | 0,01 | 0,02 | - |  |  |  |  |  |  |  |  |  |  |  |  |  |  |  |  |  |
| 27 | *M. n. zunilensis*, GUA, Solola, ENS 11250, UTA R-64857, M513 | 0,03 | 0,03 | 0,04 | 0,03 | 0,01 | 0,00 | 0,02 | 0,02 | 0,02 | 0,02 | 0,02 | 0,01 | 0,01 | 0,01 | 0,00 | 0,02 | 0,01 | 0,02 | 0,02 | 0,02 | 0,00 | 0,02 | 0,00 | 0,00 | 0,02 | 0,00 | - |  |  |  |  |  |  |  |  |  |  |  |  |  |  |  |  |
| 28 | *M. n. nigrocinctus*, HND, Gracias a Dios, LDW 12682, UF 157583 | 0,03 | 0,03 | 0,03 | 0,03 | 0,01 | 0,02 | 0,00 | 0,00 | 0,00 | 0,00 | 0,00 | 0,02 | 0,01 | 0,02 | 0,02 | 0,00 | 0,01 | 0,01 | 0,01 | 0,01 | 0,02 | 0,00 | 0,02 | 0,02 | 0,00 | 0,02 | 0,02 | - |  |  |  |  |  |  |  |  |  |  |  |  |  |  |  |
| 29 | *M. n. zunilensis*, GUA, Zacapa, MEA 860, UTA R-46525, M13 | 0,03 | 0,03 | 0,04 | 0,03 | 0,02 | 0,00 | 0,02 | 0,02 | 0,02 | 0,02 | 0,02 | 0,02 | 0,01 | 0,02 | 0,00 | 0,02 | 0,02 | 0,02 | 0,02 | 0,02 | 0,00 | 0,02 | 0,01 | 0,00 | 0,02 | 0,01 | 0,00 | 0,02 | - |  |  |  |  |  |  |  |  |  |  |  |  |  |  |
| 30 | *M. n. zunilensis*, GUA, Santa Rosa, ENS 9267, UTA R-45345, M28 | 0,03 | 0,03 | 0,04 | 0,03 | 0,01 | 0,02 | 0,01 | 0,01 | 0,01 | 0,01 | 0,01 | 0,02 | 0,01 | 0,02 | 0,02 | 0,01 | 0,01 | 0,01 | 0,01 | 0,02 | 0,02 | 0,01 | 0,01 | 0,02 | 0,01 | 0,02 | 0,02 | 0,01 | 0,02 | - |  |  |  |  |  |  |  |  |  |  |  |  |  |
| 31 | *M. n. nigrocinctus*, PAN, Panama, CH 5291, M45 | 0,05 | 0,06 | 0,05 | 0,05 | 0,06 | 0,05 | 0,05 | 0,05 | 0,05 | 0,05 | 0,05 | 0,06 | 0,06 | 0,06 | 0,05 | 0,06 | 0,06 | 0,06 | 0,06 | 0,06 | 0,05 | 0,06 | 0,06 | 0,05 | 0,06 | 0,05 | 0,05 | 0,06 | 0,05 | 0,06 | - |  |  |  |  |  |  |  |  |  |  |  |  |
| 32 | *M. n. nigrocinctus*, PAN, Panama, CH 5421, M179 | 0,06 | 0,06 | ? | 0,06 | 0,06 | 0,06 | ? | ? | ? | ? | ? | 0,06 | 0,06 | 0,06 | 0,06 | 0,06 | 0,06 | 0,07 | 0,07 | 0,06 | 0,06 | 0,07 | 0,06 | 0,06 | 0,07 | 0,06 | 0,05 | 0,06 | 0,06 | 0,06 | 0,00 | - |  |  |  |  |  |  |  |  |  |  |  |
| 33 | *M. n. nigrocinctus*, PAN, Coclé, BSFS 4591, USNM 579858 | 0,05 | 0,06 | 0,05 | 0,05 | 0,06 | 0,05 | 0,05 | 0,05 | 0,05 | 0,05 | 0,05 | 0,06 | 0,06 | 0,06 | 0,05 | 0,06 | 0,06 | 0,06 | 0,06 | 0,06 | 0,05 | 0,06 | 0,05 | 0,05 | 0,06 | 0,05 | 0,05 | 0,06 | 0,05 | 0,06 | 0,00 | 0,00 | - |  |  |  |  |  |  |  |  |  |  |
| 34 | *M. n. nigrocinctus,* PAN, Coclé, USNM FS 254011, MVUP 2032 | 0,05 | 0,06 | 0,05 | 0,05 | 0,06 | 0,05 | 0,05 | 0,05 | 0,05 | 0,05 | 0,05 | 0,06 | 0,06 | 0,06 | 0,05 | 0,06 | 0,06 | 0,06 | 0,06 | 0,06 | 0,05 | 0,06 | 0,05 | 0,05 | 0,06 | 0,05 | 0,05 | 0,06 | 0,05 | 0,06 | 0,00 | 0,00 | 0,00 | - |  |  |  |  |  |  |  |  |  |
| 35 | *M. l. nuchalis*, MEX, Oaxaca, JAC 25859, UTA R 64887, M328 | 0,04 | 0,05 | 0,04 | 0,04 | ? | 0,04 | 0,04 | 0,04 | 0,04 | 0,05 | 0,04 | ? | ? | 0,05 | 0,04 | 0,05 | 0,04 | 0,04 | 0,05 | 0,05 | 0,04 | 0,04 | ? | 0,05 | 0,05 | 0,04 | 0,04 | 0,04 | 0,04 | 0,05 | 0,05 | ? | 0,05 | 0,05 | - |  |  |  |  |  |  |  |  |
| 36 | *M. l. nuchalis*, MEX, Oaxaca, CIG 109, MZFC 32968, M896 | 0,05 | 0,05 | 0,05 | 0,05 | ? | 0,06 | 0,06 | 0,06 | 0,06 | 0,06 | 0,06 | ? | ? | 0,06 | 0,06 | 0,06 | 0,06 | 0,06 | 0,06 | 0,06 | 0,06 | 0,06 | ? | 0,06 | 0,06 | 0,06 | 0,06 | 0,06 | 0,06 | 0,06 | 0,06 | ? | 0,06 | 0,06 | 0,04 | - |  |  |  |  |  |  |  |
| 37 | *M. mosquitensis*, CRI, Limon, ASL 347, UCR 14911, M12 | 0,05 | 0,05 | 0,06 | 0,05 | 0,05 | 0,05 | 0,05 | 0,05 | 0,05 | 0,05 | 0,05 | 0,05 | 0,05 | 0,05 | 0,05 | 0,05 | 0,05 | 0,05 | 0,05 | 0,05 | 0,05 | 0,05 | 0,05 | 0,05 | 0,05 | 0,05 | 0,05 | 0,05 | 0,05 | 0,05 | 0,06 | 0,05 | 0,06 | 0,06 | 0,06 | 0,06 | - |  |  |  |  |  |  |
| 38 | *M. mosquitensis*, CRI, Cartago, MSM 781, UCR NA, M121 | 0,05 | 0,05 | 0,06 | 0,05 | 0,05 | 0,05 | 0,05 | 0,05 | 0,05 | 0,06 | 0,05 | 0,05 | 0,05 | 0,05 | 0,05 | 0,05 | 0,05 | 0,05 | 0,05 | 0,06 | 0,05 | 0,05 | 0,05 | 0,05 | 0,05 | 0,05 | 0,05 | 0,05 | 0,05 | 0,05 | 0,06 | 0,06 | 0,06 | 0,06 | 0,06 | 0,06 | 0,01 | - |  |  |  |  |  |
| 39 | *M. mosquitensis*, CRI, Limon, MSM 787, UCR NA, M23 | 0,06 | 0,06 | 0,06 | 0,06 | 0,05 | 0,06 | 0,05 | 0,05 | 0,05 | 0,05 | 0,05 | 0,05 | 0,05 | 0,05 | 0,05 | 0,06 | 0,05 | 0,06 | 0,06 | 0,06 | 0,05 | 0,06 | 0,06 | 0,05 | 0,06 | 0,06 | 0,05 | 0,06 | 0,06 | 0,06 | 0,06 | 0,06 | 0,06 | 0,06 | 0,06 | 0,06 | 0,01 | 0,01 | - |  |  |  |  |
| 40 | *M. mosquitensis*, CRI, Puntarenas, MSM NA, UCR NA, M263 | 0,05 | 0,05 | ? | 0,05 | 0,05 | 0,05 | ? | ? | ? | ? | ? | 0,05 | 0,05 | 0,05 | 0,05 | 0,05 | 0,05 | 0,06 | 0,06 | 0,06 | 0,05 | 0,06 | 0,05 | 0,05 | 0,06 | 0,06 | 0,05 | 0,05 | 0,05 | 0,05 | 0,06 | 0,06 | 0,05 | 0,05 | ? | ? | 0,01 | 0,02 | 0,02 | - |  |  |  |
| 41 | *M. mosquitensis*, CRI, Limon, MSM 788, UCR NA, M11 | 0,05 | 0,06 | ? | 0,05 | 0,05 | 0,05 | ? | ? | ? | ? | ? | 0,05 | 0,05 | 0,05 | 0,05 | 0,05 | 0,05 | 0,06 | 0,06 | 0,05 | 0,05 | 0,06 | 0,06 | 0,05 | 0,06 | 0,05 | 0,05 | 0,05 | 0,05 | 0,06 | 0,06 | 0,06 | 0,06 | 0,06 | ? | ? | 0,02 | 0,02 | 0,00 | 0,02 | - |  |  |
| 42 | *M. mosquitensis*, CRI, Puntarenas, MSM NA, UCR 13492, M20 | 0,04 | 0,05 | ? | 0,04 | 0,05 | 0,05 | ? | ? | ? | ? | ? | 0,05 | 0,05 | 0,05 | 0,04 | 0,05 | 0,05 | 0,06 | 0,05 | 0,05 | 0,04 | 0,05 | 0,05 | 0,05 | 0,05 | 0,05 | 0,05 | 0,05 | 0,05 | 0,05 | 0,05 | 0,05 | 0,05 | 0,05 | ? | ? | 0,01 | 0,02 | 0,02 | 0,00 | 0,02 | - |  |
| 43 | *M. mosquitensis*, CRI, Limon, MSM 789, UCR NA, M18 | 0,05 | 0,05 | 0,05 | 0,05 | 0,05 | 0,05 | 0,05 | 0,05 | 0,05 | 0,05 | 0,05 | 0,05 | 0,05 | 0,05 | 0,05 | 0,05 | 0,05 | 0,06 | 0,06 | 0,05 | 0,05 | 0,05 | 0,05 | 0,05 | 0,05 | 0,05 | 0,05 | 0,05 | 0,05 | 0,05 | 0,05 | 0,05 | 0,05 | 0,05 | 0,06 | 0,05 | 0,00 | 0,00 | 0,01 | 0,01 | 0,02 | 0,01 | - |
